# NMDA Receptor: An Old but Refreshed Target for Neurodegeneration

**DOI:** 10.3390/neurosci6040102

**Published:** 2025-10-08

**Authors:** Joana M. Marques, Ricardo J. Rodrigues

**Affiliations:** 1 CNC-Center for Neuroscience and Cell Biology, University of Coimbra, 3004-504 Coimbra, Portugal; joana.marques@uc.pt; 2 CiBB-Center for Innovative Biomedicine and Biotechnology, University of Coimbra, 3004-504 Coimbra, Portugal

Different neurodegenerative diseases display varying etiologies and phenotypes, reflecting region-specific neurodegeneration. Yet, despite their distinctive pathogenic mechanisms/features, diverse neurogenerative disorders also rely on common pathogenic events. One salient example is glutamate-induced neurotoxicity or excitotoxicity [1], involving an excessive and toxic Ca^2+^ influx, mainly mediated by the highly Ca^2+^-permeable NMDA receptors (NMDARs; [2,3]). This Ca^2+^ overload then leads to the activation of calpains and other proteases mediating cytoskeleton damage, reactive oxygen species generation, mitochondrial dysfunction, and subsequent neuronal apoptosis [2,3,4,5]. This NMDARs-mediated neurotoxicity was proposed to be a final pathway of neurodegeneration shared by the different neurological disorders [6], which introduced the remarkable possibility of the development of a single therapeutic strategy for all neurodegenerative diseases through the modulation of NMDAR function. The interest in NMDARs as a therapeutic target has been extended over the years to neuropsychiatric and other disorders (e.g., [7]), a topic also approached in this Special Issue in *NeuroSci* [8,9]. Naturally, NMDAR has been a long-standing potential therapeutic target and the object of intensive study.

However, NMDARs also play a key role in synaptic physiology and brain function (e.g., [10]). Hence, the challenging goal has been to find a strategy to selectively inhibit the “bad” NMDARs, while safe-guarding the critical physiological role of NMDARs. In this regard, evidence has pointed to the pro-survival action of synaptic NMDARs and a deleterious action of extrasynaptic NMDARs, activated upon excessive spillover of glutamate outside the active zone, effected by the triggering of distinct intracellular pathways [11,12,13]. Yet, the limited clinical outcome of memantine, a low-affinity non-selective antagonist of NMDARs [14], indicates that the subcellular-specific targeting of NMDARs is not sufficient. The enrichment of synaptic NMDARs with GluN2A-containing receptors and of extrasynaptic NMDARs with higher-affinity GluN2B-containing receptors [13,15,16] opened an avenue of research focusing on selectively targeting GluN2B-containing receptors to selectively arrest NMDAR-induced neurotoxicity or positively regulate GluN2A-containg receptors to restore or minimize an imbalance in subunit-specific NMDARs (for review, e.g., [17]). Nevertheless, since there is no full compartmentalization in the subcellular expression of the different subunits, subunit-specific targeting may not provide the desired efficiency in abrogating/reducing NMDAR-induced neurodegeneration, while preserving the critical physiological role of NMDARs.

Other strategies are potentially feasible, such as the control of extracellular levels of glutamate, for instance through the regulation of astrocytic function or through the indirect inhibition of the neurotoxic NMDARs by other signaling systems, whose activity is also associated with different neurodegenerative diseases. In this regard, as an example, it was shown that ATP, whose extracellular levels are also systematically increased in different neurodegenerative disorders [18], including in excitotoxic conditions [19], catalyzes glutamate-induced synaptic loss and latter neuronal death. This entails a P2Y1 receptor-driven toxic selective increase in axonal NMDARs-mediated Ca^2+^-entry and subsequent calpain-mediated axonal cytoskeleton damage, prior to any dendritic damage [19]. This study showed that P2Y1R selectively promotes the neurotoxic action of NMDAR, offering the possibility to selectively inhibit an NMDAR-mediated neurotoxic action by targeting another signaling system, avoiding any impact on physiological NMDAR function.

The ability to solely inhibit the damaging activity of NMDARs may also arise from the possibility of developing NMDAR allosteric modulators to regulate particular NMDAR subpopulations, also in a cellular or region-specific manner, or by targeting heterocomplexes formed by NMDARs and other receptors or proteins that may confer the NMDARs with neurotoxic action. This would offer natural selectivity for a therapeutic approach that could avoid the adverse effects from interfering with NMDARs. Such a possibility was recently supported by the remarkable demonstration of the existence of NMDAR-TRPM4 (transient receptor potential cation channel subfamily M member 4) death complex [20]. It was found that the NMDAR subunits, both GluN2A and GluN2B, but not GluN1, form a complex with TRPM4 selectively at extrasynaptic sites. This confers neurotoxicity to the extrasynaptic NMDARs previously observed, since the inhibition of its formation using interaction interface inhibitors provided robust neuroprotection against excitotoxicity both in vitro and in vivo [20]. Importantly, the inhibition of the formation of the NMDAR/TRPM4 complex does not interfere with physiological synaptic and extrasynaptic NMDAR signaling [20]. This finding clearly opens the possibility to selectively abrogate neurotoxic NMDARs without adverse effects, which will hopefully constitute a major advance in the therapeutics of neurodegenerative diseases [21]. Accordingly, the inhibition of the NMDAR/TRPM4 death complex formation was already shown to provide robust neuroprotection in animal models of amyotrophic lateral sclerosis [22,23] and more recently in Alzheimer’s disease [24], which most likely will be extended to other neurodegenerative disorders. Hopefully, this will soon introduce efficient therapeutics for neurodegenerative disorders.

From an academic point of view, there are also interesting questions that remain to be clarified. Although there is an upregulation of this NMDAR/TRPM4 complex in pathological conditions, it was identified in physiological conditions [24]. Does it have a physiological purpose? The inhibition of its formation had no impact in memory-associated tasks [24]. Is its primary function to induce programed/physiological cell death? Is it formed during development or only in adulthood? Does it increase with aging? Whatever the answers, the identification of this NMDAR/TRPM4 complex undoubtedly constitutes a major advance in our comprehension of NMDARs and for the development of a strategy to arrest or at least delay neurodegenerative disorders, an unmet medical need.

## References

[1] Olney J.W. (1969). Brain lesions, obesity, and other disturbances in mice treated with monosodium glutamate. Science.

[2] Choi D.W. (1994). Calcium and excitotoxic neuronal injury. Ann. N. Y. Acad. Sci..

[3] Rothman S.M., Olney J.W. (1995). Excitotoxicity and the NMDA receptor–still lethal after eight years. Trends Neurosci..

[4] Vanderklish P.W., Bahr B.A. (2000). The pathogenic activation of calpain: A marker and mediator of cellular toxicity and disease states. Int. J. Exp. Pathol..

[5] Dawson V.L., Dawson T.M. (2004). Deadly conversations: Nuclear-mitochondrial cross-talk. J. Bioenerg. Biomembr..

[6] Lipton S.A., Rosenberg P.A. (1994). Excitatory amino acids as a final common pathway for neurologic disorders. N. Engl. J. Med..

[7] Beaurain M., Salabert A.S., Payoux P., Gras E., Talmont F. (2024). NMDA receptors: Distribution, role, and insights into neuropsychiatric disorders. Pharmaceuticals.

[8] Shangase K.B., Magwai T., Oginga F.O., Xulu K.R., Mpofana T. (2022). Effectiveness of double-hit model (post-weaning social isolation and NMDA receptor antagonist) in the development of schizophrenic like symptoms on rodents: A protocol for a systematic review. NeuroSci.

[9] Thomas A., Chambers R.A. (2025). Ketamine’s therapeutic role in substance use disorders: A narrative review. NeuroSci.

[10] Paoletti P., Bellone C., Zhou Q. (2013). NMDA receptor subunit diversity: Impact on receptor properties, synaptic plasticity and disease. Nat. Rev. Neurosci..

[11] Sattler R., Xiong Z., Lu W.Y., Macdonald J.F., Tymianski M. (1999). Distinct roles of synaptic and extrasynaptic NMDA receptors in excitotoxicity. J. Neurosci..

[12] Hardingham G.E., Fukunaga Y., Bading H. (2002). Extrasynaptic NMDARs oppose synaptic NMDARs by triggering CREB shut-off and cell death pathways. Nat. Neurosci..

[13] Hardingham G.E., Bading H. (2010). Synaptic versus extrasynaptic NMDA receptor signalling: Implications for neurodegenerative disorders. Nat. Rev. Neurosci..

[14] Johnson J.W., Kotermanski S.E. (2006). Mechanism of action of memantine. Curr. Opin. Pharmacol..

[15] Fellin T., Pascual O., Gobbo S., Pozzan T., Haydon P.G., Carmignoto G. (2004). 2004 Neuronal synchrony mediated by astrocytic glutamate through activation of extrasynaptic NMDA receptors. Neuron.

[16] Papouin T., Oliet S.H. (2014). Organization, control and function of extrasynaptic NMDA receptors. Philos. Trans. R. Soc. Lond. B. Biol. Sci..

[17] Zhang K., Wen M., Nan X., Zhao S., Li H., Ai Y., Zhu H. (2025). NMDA receptors in neurodegenerative diseases: Mechanisms and emerging therapeutic strategies. Front. Aging Neurosci..

[18] Rodrigues R.J., Figueira A.S., Marques J.M. (2022). P2Y1 receptor as a catalyst of brain neurodegeneration. NeuroSci.

[19] Simões A.P., Silva C.G., Marques J.M., Pochmann D., Porciúncula L.O., Ferreira S., Oses J.P., Beleza R.O., Real J.I., Köfalvi A. (2018). Glutamate-induced and NMDA receptor-mediated neurodegeneration entails P2Y1 receptor activation. Cell Death Dis..

[20] Yan J., Bengtson C.P., Buchthal B., Hagenston A.M., Bading H. (2020). Coupling of NMDA receptors and TRPM4 guides discovery of unconventional neuroprotectants. Science.

[21] Yan J., Bading H. (2023). The disruption of NMDAR/TRPM4 death signaling with TwinF interface inhibitors: A new pharmacological principle for neuroprotection. Pharmaceuticals.

[22] Wang Y.M., Yan J., Williams S.K., Fairless R., Bading H. (2024). TwinF interface inhibitor FP802 prevents retinal ganglion cell loss in a mouse model of amyotrophic lateral sclerosis. Acta Neuropathol Commun..

[23] Yan J., Wang Y.M., Hellwig A., Bading H. (2024). TwinF interface inhibitor FP802 stops loss of motor neurons and mitigates disease progression in a mouse model of ALS. Cell Rep. Med..

[24] Yan J., Yang X., Li G., Ramirez O.A., Hagenston A.M., Chen Z.Y., Bading H. (2025). The NMDAR/TRPM4 death complex is a major promoter of disease progression in the 5xFAD mouse model of Alzheimer’s disease. Mol. Psychiatry..

